# Lipidomic approach for stratification of acute myeloid leukemia patients

**DOI:** 10.1371/journal.pone.0168781

**Published:** 2017-02-16

**Authors:** Adam Stefanko, Christian Thiede, Gerhard Ehninger, Kai Simons, Michal Grzybek

**Affiliations:** 1 Max Planck Institute of Molecular Cell Biology and Genetics, Dresden, Germany; 2 Medical Clinic and Polyclinic I, University Hospital TU Dresden, Dresden, Germany; 3 Lipotype GmbH, Dresden, Germany; 4 Paul Langerhans Institute Dresden of the Helmholtz Centre Munich at the University Clinic Carl Gustav Carus, TU Dresden, Dresden, Germany; 5 German Center for Diabetes Research (DZD e.V.), Neuherberg, Germany; Stony Brook University, UNITED STATES

## Abstract

The pathogenesis and progression of many tumors, including hematologic malignancies is highly dependent on enhanced lipogenesis. De novo fatty-acid synthesis permits accelerated proliferation of tumor cells by providing membrane components but these may also alter physicochemical properties of lipid bilayers, which can impact signaling or even increase drug resistance in cancer cells. Cancer type-specific lipid profiles would permit us to monitor and interpret actual effects of lipid changes, potential fingerprints of individual tumors to be explored as diagnostic markers. We have used the shotgun MS approach to identify lipid patterns in different types of acute myeloid leukemia (AML) patients that either show no karyotype change or belong to t(8;21) or inv16 types. Differences in lipidomes of t(8;21) and inv(16) patients, as compared to AML patients without karyotype change, presented mostly as substantial modulation of ceramide/sphingolipid synthesis. Furthermore, between the t(8;21) and all other patients we observed significant changes in physicochemical membrane properties. These were related to a marked alteration in lipid saturation levels. The discovered differences in lipid profiles of various AML types improve our understanding of the pathobiochemical pathways involved and may serve in the development of diagnostic tools.

## Introduction

Acute Myeloid Leukemia (AML) is a clonal bone marrow disorder resulting from diverse phenotypic and genetic alterations in the differentiation of hematopoietic stem cells and causing excessive proliferation and accumulation of abnormal immature leukemic neoplastic cells, called blasts[1–4]. The major causes of AML are deregulation in one or more of the numerous components of signaling networks that control cell growth, either by gain-of-function mutation or overexpression[5, 6]. Most efforts towards molecular characterization of AML have focused on genome, transcriptome or proteome changes [7–12] while evidence is growing that neoplastic pathogenesis and progression also involve, and might even be accelerated by, changes in cellular lipidomes[13–19]. In fact, one of the hallmarks of many tumors including hematologic malignancies is enhanced lipogenesis, arising from increased activities of fatty acid biosynthetic enzymes (Acc1, Fasn and Scd1)[20–23], necessary for accelerated growth. Furthermore, synthesis of mono-unsaturated fatty acids may have added benefit, since oleate can protect against saturated fatty acid toxicity and cellular stress[18, 22, 24, 25]. Thus, Acc1, Fasn and Scd1 have been identified as plausible targets for cancer therapy[14, 18, 20–23].

One of the lipid classes associated with cancer pathogenesis are the sphingolipids with their metabolites such as ceramide, hexosylceramide and sphingosine-1-phosphate, all of which act as effector molecules[15]. In particular, ceramides are collectively involved in the regulation of cancer-cell growth, differentiation, senescence and apoptosis[26–28]. Conversion of ceramide to glucosylceramide contributes to multidrug resistance of several human cancers, including leukemia, breast cancer, melanoma and neuroblastoma[17, 29–31]. Of equal importance, sphingolipids are structural lipids that tune membrane fluidity and subdomain structure of the lipid bilayer, especially lipid rafts[32]. These nanodomains act as regulatory platforms for a number of growth factor receptors e.g. EGFR, c-kit, VEGFR etc. controlling the initiation of signaling cascades in cell growth and differentiation[33, 34]. Therefore, deregulation of lipid metabolism threatens to perturb lateral membrane organization. Mechanisms that govern homeostasis within the enormous heterogeneity of distinct lipid species are largely unknown. Notably, metabolic transitions affecting lipid metabolism can prompt lipidome-wide ‘concentration waves’, in turn triggering compensatory metabolic responses[35, 36]. There is a growing interest in lipidome changes during pathogenesis, also in the hope of establishing unique cellular fingerprints useful as diagnostic markers.

The purpose of this study was to explore the lipidomic profiles of AML patients. We focused on three subsets of patients with specific clinical and biological AML characteristics: translocation t(8;21), inversion inv16 and patients with normal karyotype (AML-nk). Chromosomal abnormalities are detected in approximately 60% of newly diagnosed AML patients, among which t(8;21) and inv16 are the most common. They affect the expression of the Core Binding Factor (CBF)—a heterodimeric transcription factor controlling key programs in cell survival[37–39], at least in part by regulating sphingolipid metabolism [40, 41]. Both t(8;21) and inv16 are usually associated with good prognosis and survival. The ‘normal karyotype’ AML group is very heterogeneous as it emanates from diverse factors related to aberrant signal transduction pathways leading to uncontrolled growth and inhibition of apoptosis[2, 6, 42]. Moreover, these changes are often associated with increased activity of multidrug resistance proteins and the renewal of leukemia stem cells[30, 31].

Here, applying shotgun mass spectrometry we analyzed the cellular lipidomes of various AML types and identified salient differences. Major discordancies concerned the sphingolipids and their metabolites. We also observed pronounced shifts in membrane fluidity among the studied AML types. The recognized lipid patterns may guide the identification of druggable metabolic pathways for personalized anti-neoplastic therapies.

## Materials and methods

### Sample collection

All AML samples investigated in this study were bone marrow aspirates (anticoagulant lithium heparin) collected at the time of diagnosis. The blast fraction was enriched by density gradient centrifugation (density 1.077, BIOCOLL separating solution, Biochrom, Berlin Germany). Enriched samples were aliquoted (1–2 x10^6^ cells/ ml), frozen viably in medium containing DMSO and fetal calf serum (FCS) and subsequently stored until analysis in liquid nitrogen. Cytogenetic and molecular analysis followed established methods as described [43, 44]. Patients were enrolled in prospective treatment protocols of the Study Alliance Leukemia (SAL). All patients gave written informed consent making available their material and data for scientific purposes. The studies were approved by the ethical board of the Medical Faculty TU Dresden (EK 98032010). Full description of patients can be found in **Table 1**.

**Table 1:** Description of patients.

ID	karyotype	sex	age	*FLT3*-ITD mutation	*FLT3*-ITD ratio		*NPM1* mutation	*KIT* mutation	first complete remission achieved	relapse after complete remission
1	normal	f	81	N	NA		Y	NA	N	N
2	normal	m	59	Y	0.92		Y	NA	Y	Y
3	normal	f	62	Y	0.91		Y	NA	N	N
4	normal	f	72	Y	5.9		Y	NA	N	N
5	normal	f	72	N	NA		Y	NA	Y	Y
6	normal	m	63	Y	NA		Y	NA	Y	N
7	inv16	f	53	N	NA		N	N	N	N
8	inv16	f	30	N	NA		N	N	Y	Y
9	inv16	m	28	N	NA		N	Exon 8	Y	Y
10	inv16	m	36	N	NA		N	Exon 8	Y	N
11	inv16	m	41	N	NA		N	N	Y	Y
12	t(8;21)	m	37	N	NA		N	N	Y	N
13	t(8;21)	f	26	N	NA		N	D816V/H	Y	N
14	t(8;21)	m	41	N	NA		N	N	Y	N
15	t(8;21)	m	53	N	NA		N	D816V	Y	N
16	t(8;21)	f	34	N	NA		N	N	Y	Y

F—female; m—male; NA-no information; FLT3-ITD—FMS-like tyrosine kinase 3 internal tandem duplication; NPM1—Nucleophosmin 1; N—no; Y—yes

### Sample preparation

Synthetic lipid standards: cholesterol (Chol-d6) (deuterated), sterol ester (CE 20:0), triacylglycerol (TAG 17:0–17:0–17:0), diacylglycerols (DAG 17:0–17:0), phosphatidic acid (PA 17:0–17:0), phosphatidylcholine (PC 17:0–17:0), phosphatidylethanolamine (PE 17:0–17:0), phosphatidylglycerol (PG 17:0–17:0), phosphatidylserines (PS 17:0–17:0), phosphatidylinositol (PI 16:0–16:0), lysoglycerophospholipids (LPA 17:0, LPC 12:0, LPE 17:1, LPS 17:1, LPI 17:1), sphingomyelin (SM 18:1;2–12:0) and ceramides (Cer 18:1;2–17:0, GlcCer 18:1;2–12:0, LacCer 18:1;2–12:0) were purchased from Avanti Polar Lipids. Total protein concentration of leukemic cells was estimated using the microBCA Protein Assay Kit (Thermo Scientific). Aliquots of cells equivalent to 20–25 μg of protein that correspond to ~250 000 cells, were transferred into 2 ml tubes (Eppendorf AG), pelleted (1000g, 5’) washed twice with phosphate-buffered saline and spiked with 10 μl internal lipid standards mixture dissolved in methanol. All samples were subjected to two-step lipid extraction with 750 μl of 10:1 chloroform:methanol (C/M) (v/v) followed by a 2:1 C/M mixture at 4°C. Lipid extracts were collected from organic phases and evaporated under vacuum. Dry lipid extracts were immediately re-dissolved in 100 μl of chloroform/methanol 1:2 (v/v) and injected into the mass spectrometer Q-Exactive (Thermo Fisher Scientific) equipped with an electrospray ion source—ESI TriVersa Nanomate (Advion Biosciences). Three independent runs were done for each lipid extract.

### Shotgun lipidomics analysis and data processing

Lipid extracts (7.5 μl) from the first extraction step were diluted in 13 mM ammonium acetate in propanol (10 μl) to achieve the final solvent composition of 7.5 mM ammonium acetate in chloroform/methanol/propanol 1:2:4 (v/v). Lipid extracts from the second extraction step were dissolved in 10 μl of 0.01% methylamine as ion modifier and subjected to negative mode MS analysis. Lipid extracts from the 10:1 organic phase were analyzed with polarity switching in both positive and negative ion mode, the 2:1 extracts in negative mode only. Positive ion mode analysis was performed using FTMS with target resolution of 280,000 at m/z 200 and MS/MS with 17,500 at m/z 200 to monitor CE, DAG and TAG species. Negative FTMS mode was chosen to monitor lyso-phospholipids (LPG, LPS, LPI, LPS, LPC, LPE) and combined with tandem MS/MS for selected phospholipids (PC, PC O-, PE, PE O-, PS, PA, PI, PG), sphingolipids (SM, Cer, Hex-Cer, diHex-Cer), globosides (Gb3 and Gb4) and ganglioside GM3. The same, targeted resolution settings were used for both polarities. Cholesterol was measured separately after chemical acetylation[45]. Briefly, 20 μl of 10:1 lipid extract was dried down and 75 μl of acetyl chloride in 1:2 C/M (v/v) added. After an hour incubation samples were dried down, re-dissolved in 50 μl C/M 1:2 and analyzed in positive ion mode with similar settings as described above. The molarity of lipid species was determined based on spiked-in, internal quantitative standards.

Samples were infused into the Q-Exactive instrument TriVersa NanoMate ESI source. Data acquisition was performed in both positive and negative mode with polarity switching where MS was used for quantification and MS/MS for fatty acid and lipid precursor identification. Data were analyzed and de-convoluted by LipidXplorer software. Data visualization and normalization (pmol, mol%) calculations were performed in Microsoft Excel, GraphPad Prism 6.0 and SIMCA 14.0.

### GP measurements

For fluidity analysis, dried lipids were rehydrated in 150 mM NaCl, 25 mM HEPES pH 7.25. The resulting liposomes were subsequently subjected to 10 freezing/thawing cycles (liquid nitrogen/37°C) and extruded through 100 nm polycarbonate filters. Finally, vesicles were stained (15 min) with 2 μM C-laurdan. Fluorescence emission spectra were recorded (Ex. 385 nm, Em. 400–550 nm) and analyzed as described in Kaiser et al.[46].

## Results

### Lipidome analysis reveals significant differences among the AML subtypes

Total leukocyte fractions containing 250 000 cells, corresponding to approximately 20–25 μg of total protein content, were subjected to two-step lipid extraction, MS lipidomics and GP measurements. Shotgun lipidomics MS and MS/MS designated acquisition routine and Lipotype proprietary lipidomics software enabled profiling of more than 400 unique lipid species of 25 analyzed lipid classes (for details see S1 Table).

**Fig 1:**
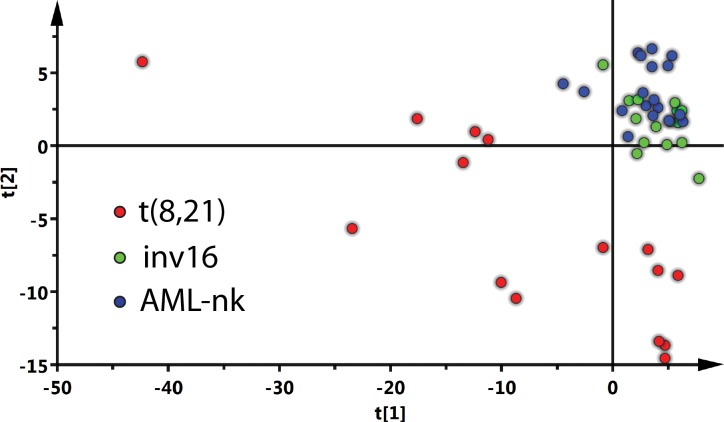
Score plots of multivariate analysis by PCA. The t(8;21) samples are separated from the inv16 and AML-nk samples. Each patient sample was measured in triplicates and each point on the plot represents an individual measurement. The calculated sum of squares was 0.253 and 0.094 for the first and second component, respectively. The analysis was performed using SIMCA 14.0 software.

Lipidomics data were first normalized to internal standards and transformed to mol%. Multivariate statistical data analysis was applied to systematize and better understand the recorded lipidome variations. Dimension reduction and data visualization followed Principal Component Analysis (PCA) with Pareto scaling (SIMCA software). This statistical tool emphasizes variation and highlights robust patterns in a dataset. Subsequently, a two-dimensional score plot was generated (significance of the principal components based on the explained sum of squares was 0.253 and 0.094 for the first and second principal component respectively) (Fig 1), revealing t(8;21) samples clearly separated from inv16 and AML-nk patient samples—the latter clustered together. This suggested substantial differences between the lipidomes of t(8;21) and the other AML types.

**Fig 2:**
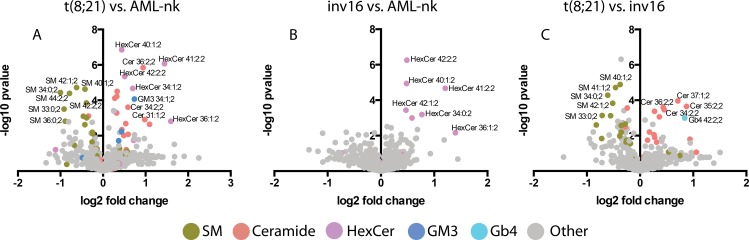
Comparison of lipid profiles of various AML types. Each point on the volcano graph represents a single lipid specie. The analysis was performed using GraphPad PRISM 6.0.

Next, lipid species differences between the AML types were analyzed. Briefly, lipid fold change followed by multiple t-tests analysis identified unique, species with the most pronounced deviations (Fig 2). The major difference between t(8;21) and AML-nk involved ceramide backbone-containing lipids: SM, Cer, Hex-Cer and GM3 ganglioside (Fig 2A). Decrease of SM in t(8;21) was correlated with an increase in the levels of ceramides (up to 3-fold), Hex-Cer (up to 5-fold), DiHex-Cer (up to 3-fold) and GM3 (up to 15-fold) suggesting a shift towards glycosphingolipid synthesis (S1 Table). Unfortunately GM3 derivatives are currently not accessible to quantitative shotgun MS. Similarly, the comparison of groups t(8;21) and inv16 (Fig 2B) mostly registered expression profile changes affecting sphingolipid pathways. We observed a decrease in SM levels and increased ceramide synthesis (Fig 2C), suggesting that this type of leukemic cells might be more prone to apoptosis[26–28]. Despite the fact that inv16 and AML-nk patients represent a mixed population in the PCA plot (Fig 1), direct comparison by orthogonal partial least-square displacement analysis brings to light considerable differences (p<0.001) between their lipidomes. The most prominent change was the increased abundance of various Hex-Cer species in the inv16 samples (Fig 2C). These lipids are metabolic precursors of other glycolipids, but were also ascribed an important role in multidrug resistance[17, 29, 31]. Inv16 is associated with a specific accumulation of one particular lipid class, rather than a shift in glycosphingolipid metabolism, as in the case of t(8;21) cells.

### Measurement of generalized polarization index reveals dramatic changes in membrane fluidity in t(8;21) cells

**Fig 3:**
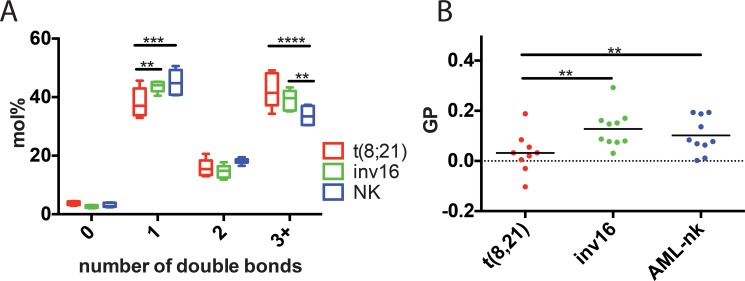
Lipid features from extracted AML samples. (A) Saturation index of all lipids measured with shotgun MS. The analysis revealed significant changes of the mono- and polyunsaturated fatty acids in t(8;21) samples. (B) GP index of liposomes prepared from lipid extracts of various AML samples. GP is a measure of membrane order (higher GP equals more ordered membranes). t(8;21) samples exhibit lower GP values, indicating higher membrane fluidity.

Activation of the lipogenic pathway at early stages of cancer pathogenesis is critical for proliferation of various types of tumors including hematologic malignancies[21, 23]. The majority of newly synthesized fatty acids in cancer cells are converted predominantly to membrane phospholipids. Specifically, the proportion of monounsaturated and polyunsaturated fatty acids in the major phospholipids was reported to be significantly higher in cancer compared to noncancerous tissues[47]. Here we compared each type of leukemia for differences in lipid saturation levels. A marked difference affected t(8;21) leukemia, a significant shift towards polyunsaturated at the cost of monounsaturated fatty acids (Fig 3A). This observation was specific to membrane lipids (phospholipids + sphingolipids), which comprise roughly 80% of the total measured lipids, but not to storage lipids (TAG, DAG, SE) (S1 Fig).

Fatty acid saturation and length plus cholesterol content are the three major determinants of membrane fluidity. Having observed a significant shift of fatty acid saturation but not of cholesterol abundance or fatty acid length (Figs 3 and S1) we expected a substantial change in membrane fluidity of t(8;21) compared to the other AML types. Membrane fluidity (General Polarization Index, GP) was measured on small unilamellar vesicles prepared from the extracted lipids, using the fluorescent probe c-laurdan, as described [48]. Levels of unsaturation and GP values were closely correlated, confirming the greater fluidity of t(8;21) membranes. These findings underscore the importance of precisely measuring lipid and desaturation levels in cancer cells to resolve aberrantly regulated metabolic pathways and, ultimately, therapeutic targets.

## Discussion

Similar to embryonic cells, cancer cells are highly dependent on de novo lipogenesis for their proliferation, and lipogenesis is activated at a relatively early stage in various types of tumors[18, 21, 49]. Moreover, there seems to be a correlation between fatty acid synthase expression, which is greatly elevated in metastatic tumors, increased membrane fluidity and decreased patient survival[50–53]. Proliferating cancer cells predominantly convert the majority of newly synthesized fatty acids to phospholipids for membrane incorporation [18]. Changes in lipid metabolism disequilibrating saturation levels of membrane fatty acids influence membrane fluidity and structure and composition of membrane domains (rafts) where many critical signaling cascades originate. In consequence, aberrant signal transduction may enhance the proliferation and survival of hematopoietic progenitor cells[2, 6]. An additional layer of complexity arises as a consequence of metabolic interconversion of one lipid to another—e.g. of PI(4,5)P2 to DAG or DAG/PC to PA—that impact signaling[54, 55]. Similarly, many pathways of sphingolipid metabolism constitute an interconnected network wherein individual levels and the balance of bioactive molecules can modulate cancer initiation, progression or treatment response. For example, ceramide hydrolysis produces sphingosine, the phosphorylation of which yields sphingosine-1-phosphate that regulates cell growth and suppresses programmed cell death[56–58]. Thus the difference in lipid metabolism between tumors and normal tissues renders lipogenic pathways attractive but so far only moderately exploited targets for the development of anti-cancer therapies [23, 59].

To evaluate the suitability of lipidomics for discriminating various types of cancer cells we analyzed the lipid profiles of AML cells of patients with (t(8;21), inv16) and without karyotype disorders (AML-nk). Importantly, in both t(8;21) and inv16 AML the karyotype changes result in deregulation of CBF expression, a factor that controls the expression of genes involved in sphingolipid metabolism[40, 41]. CBF overexpression has been shown to reduce intracellular long-chain ceramides in NIH3T3 fibroblasts and to elevate extracellular sphingosine-1-phosphate levels[40]. On the other hand, CBFβ-silenced cells exhibited substantial increases in ceramide species and decreases in lactosylceramides that correlated with enhanced cell death[41]. Here for the first time we demonstrate quantitative lipidome changes of cells with disorders in CBF expression. Indeed the major differences discovered between the various AML types affect the sphingolipids and ceramides. Interestingly, although both t(8;21) and inv16 share similarities (deregulation of CBF expression), significant differences in sphingolipid metabolism remain. Translocation t(8;21) unlike inv16 and AML-nk, is accompanied by substantial decrease of sphingomyelin (Fig 2), probably, as shown by others, due to decreased expression of SGMS1 and SGMS2[7], which detours ceramides to glycosphingolipid synthesis. In inv16 samples sphingolipid metabolism appears more at equilibrium and only hexosylceramides are specifically accumulated (Fig 2C), reported as a multidrug resistance phenotype in many cancer cells[17, 29–31]. The observed pattern is particularly revealing, as in t(8;21) and inv16 patients sensitive to the drugs ABT737 (Bcl-2 inhibitor) and BV6 (antagonist of IAP, ‘inhibitor of apoptosis proteins’), genes responsible for ceramide synthesis were upregulated, in contrast to patients resistant to these drugs[7]. Enzyme expression changes of sphingolipid metabolism in several cancers are consistent with the emerging tumor suppressor functions of ceramide. Our data provide evidence that ceramide levels significantly differing between patients, even those bearing similar genetic perturbations, might directly correlate with these patients’ susceptibility to chemotherapy.

Among all studied groups, the t(8;21) patients presented the highest membrane fluidity, predominantly due to increased unsaturation of membrane lipid fatty acid moieties. Elevated membrane fluidity has been associated with enhanced cell growth and proliferation in many cancers[60–62] as well as shown to modulate certain receptor tyrosine kinase proteins, resulting in aberrant signaling[63–66]. On the other hand the shift towards increased levels of saturated and monounsaturated phospholipids may protect cancer cells from oxidative damage by reducing lipid peroxidation[14, 18]. In line with this, it has been suggested of membrane active drugs that their ability to alter membrane fluidity correlates with pharmacological activity[67, 68].

The observed changes in membrane fluidity in t(8,21) samples might, to a lesser extent, be the consequence of lower sphingomyelin concentration. Yet, this appears to be compensated by the increase in glycosphingolipid (GSL) content (S1 Fig), making a dramatic effect on membrane fluidity unlikely. Interestingly however, the changes in t(8,21) samples in the ratio of SM and GSL might critically alter the physicochemical properties of rafts with important sequels for signal transduction pathways. Glycosphingolipids were among the first discovered tumor-associated antigens and are now appreciated as function modulators of several growth factor receptors that govern cell growth and differentiation [69, 70]. Although the current approach does not allow assaying of higher glycolipids (with more than three sugar residues), the observable ones (Hex-Cer, DiHex-Cer and GM3) indicated a significant shift in sphingomyelin-glycosphingolipid metabolism. Therefore, one can clearly expect significant, purely lipid-induced responses in signal transduction pathways not brought about by changes in expression levels or gain-of-function mutations in the components of the signaling cascades. The challenge for the future will be to unravel how, through modulation of the growth signaling cascades, individual differences in sphingolipid metabolism stimulate cancer development and progression. As shown here, quantification of cellular lipids, down to single species level allowed us to distinguish AML-type-specific lipid signatures. Such procedures pave the way towards new diagnostics and prognostics and should be integrated into a systemic approach towards the mechanistic understanding of cancer and the optimization of therapeutical targets.

## Conclusion

Mass spectrometry-driven quantitative analysis of cellular lipids, especially the so-called shotgun lipidomics technique is increasingly being explored for its potential as a clinical diagnostic tool. A major benefit of this technology is that hundreds of lipid species can be directly identified and accurately quantified in a relatively short analysis time[71–73]. This work demonstrates that lipidomic profiling of acute myeloid leukemia cells supports the stratification of hematological malignancies. The observed imbalances of lipid synthesis pathways might directly contribute to disease progression.

## Supporting information

S1 Fig (A) Lipid features (fatty acid saturation and length) measured with shotgun MS for “membrane” (PC, PC-O, PE, PE-O, PS, PI, PG, PA, SM, Cer, HexCer, DiHexCer, GM3, Gb3, Gb4) and “storage” lipids (SE, TAG, DAG). (B) Lipid profile of sphingolipids measured in various AML samples.
(PDF)

S1 Table Lipidomic analysis of AML samples. Mol% values of individual lipid species determined with shotgun MS for particular patient samples. Each sample was measured in three independent runs.
(XLSX)
