# Supplementary figures and images for: Lipidomic approach for stratification of acute myeloid leukemia patients
Source: PLoS One. 2017 Feb 16;12(2):e0168781. doi: 10.1371/journal.pone.0168781 (PMC5313223; doi:10.1371/journal.pone.0168781)

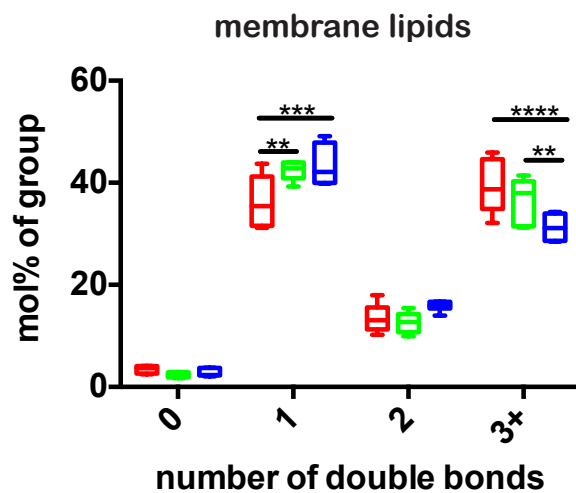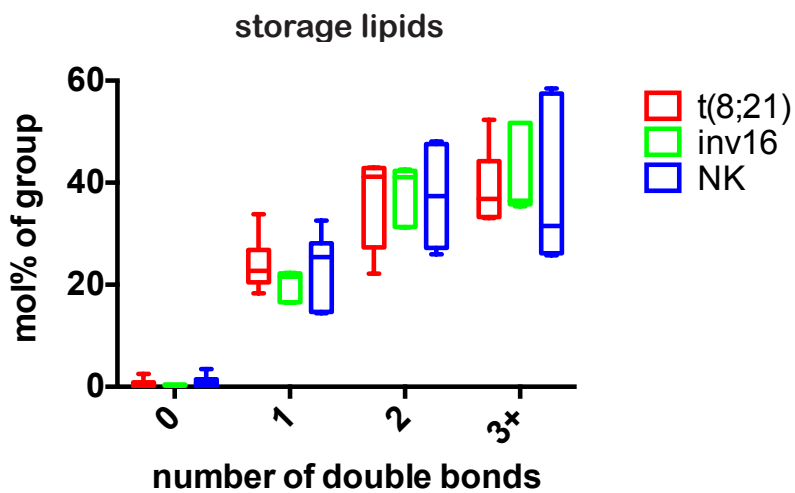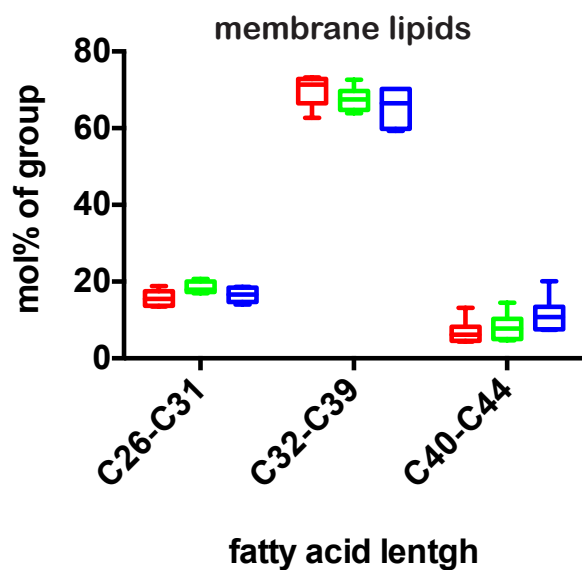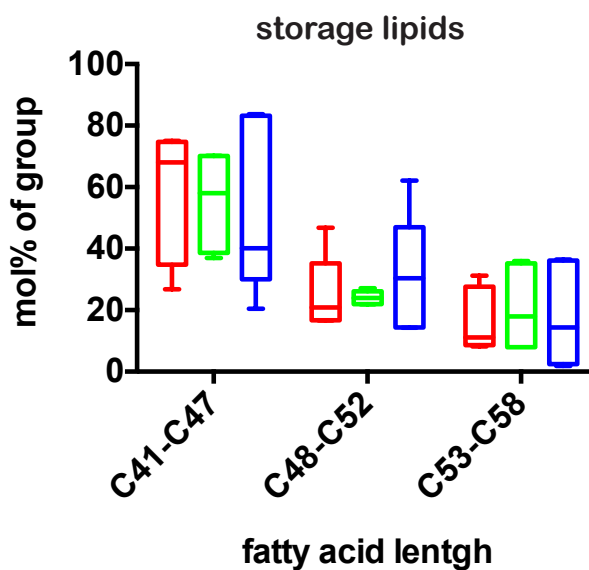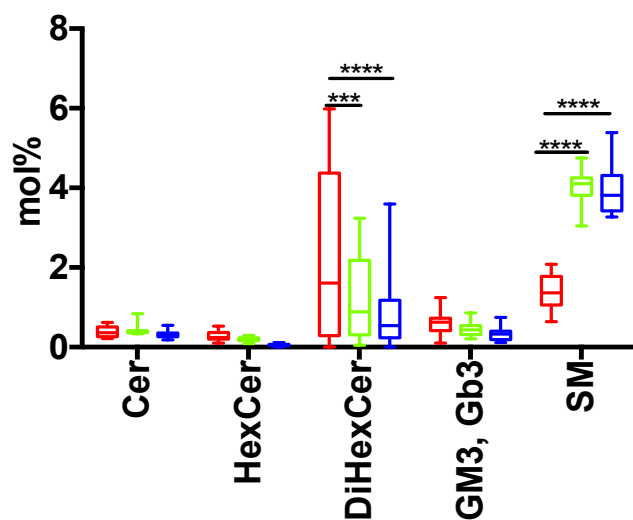

Supplement: S1 Fig — (A) Lipid features (fatty acid saturation and length) measured with shotgun MS for “membrane” (PC, PC-O, PE, PE-O, PS, PI, PG, PA, SM, Cer, HexCer, DiHexCer, GM3, Gb3, Gb4) and “storage” lipids (SE, TAG, DAG). (B) Lipid profile of sphingolipids measured in various AML samples. (PDF) [file pone.0168781.s001.pdf]
